# The less healthy urban population: income-related health inequality in China

**DOI:** 10.1186/1471-2458-12-804

**Published:** 2012-09-18

**Authors:** Wei Yang, Panos Kanavos

**Affiliations:** 1 Department of Social Policy, LSE Health London School of Economics and Political Science, Houghton Street, London, WC2A 2AE, UK

**Keywords:** Health inequality, Adult health, Urban and rural disparity, China

## Abstract

**Background:**

Health inequality has been recognized as a problem all over the world. In China, the poor usually have less access to healthcare than the better-off, despite having higher levels of need. Since the proportion of the Chinese population living in urban areas increased tremendously with the urbanization movements, attention has been paid to the association between urban/rural residence and population health. It is important to understand the variation in health across income groups, and in particular to take into account the effects of urban/rural residence on the degree of income-related health inequalities.

**Methods:**

This paper empirically assesses the magnitude of rural/urban disparities in income-related adult health status, i.e., self-assessed health (SAH) and physical activity limitation, using Concentration Indices. It then uses decomposition methods to unravel the causes of inequalities and their variations across urban and rural populations. Data from the China Health and Nutrition Survey (CHNS) 2006 are used.

**Results:**

The study finds that the poor are less likely to report their health status as “excellent or good” and are more likely to have physical activity limitation. Such inequality is more pronounced for the urban population than for the rural population. Results from the decomposition analysis suggest that, for the urban population, 76.47 per cent to 79.07 per cent of inequalities are driven by non-demographic/socioeconomic-related factors, among which income, job status and educational level are the most important factors. For the rural population, 48.19 per cent to 77.78 per cent of inequalities are driven by non-demographic factors. Income and educational attainment appear to have a prominent influence on inequality.

**Conclusion:**

The findings suggest that policy targeting the poor, especially the urban poor, is needed in order to reduce health inequality.

## Background

Health inequality has been recognized as a problem all over the world. A large and growing body of research has examined the hypothesis that the individual’s health is shaped not just by the absolute level of resources available to them, but also by the level of resources available to them relative to others in their cohort or community [1-4]. Inequality in income has grown at a startling pace in the last 25 years in China and scholars generally agree that disparities in income are considered to be one important factor leading to inequality in health [5-8]. In China, studies show that the poor usually have less access to healthcare than the better-off, despite having higher levels of need. Notwithstanding their lower levels of utilization, the poor often spend more on healthcare as a share of their income than the better-off [9-11].

As the proportion of the Chinese population living in urban areas has grown tremendously with the urbanization movements in China, attention has been paid to the association between urban/rural residence and the health of the population. Earlier studies found that, in general, health outcomes were better in urban China. For instance, the prevalence of child stunting was much lower in urban than in rural China [12]. The rural elderly were more likely to experience functional limitation than the urban elderly, and were less likely to survive a two-year follow-up period [13]. However, earlier studies mainly focused on comparisons between average health of urban and rural populations, and most were descriptive. Reports on income-related differences in health between urban and rural populations are relatively rare [9-11]. It is critically important to understand the distribution of health in these areas, as unequal distribution may adversely affect the labor supply and productivity of the less well-off households, hence exacerbating income-related health inequality.

Interestingly, studies in this field have demonstrated different findings. Chen et al. [12] examined the issue of regional disparity in child malnutrition in China, and found that rural children were more likely to be stunted than their urban counterparts. Similar results were demonstrated in another study. The study suggested that the effect of socioeconomic status on health was stronger for people born in the 1930s and before, and weaker for those born in the 1940s and after. This pattern was more pronounced in rural populations [14]. However, findings are not always consistent. Van de Poel et al. [15] explored some aspects of the relationship between the distribution of diseases and urbanization in China. Their study found that urban residents were more likely to suffer from non-communicable diseases, and that urbanization had been proven to impose a penalty on perceived health in China. In a study examining child health in 47 developing countries, Van de Poel et al. [16] found that the urban poor actually had higher rates of stunting and mortality than their rural counterparts. The findings implied that there was a need for programs that target the urban poor, and that this was becoming more necessary as the size of the urban population grew.

The study of health inequality in China is timely and important. This article follows Erreygers, Wagstaff, van Doorslaer and O'Donnell in using Concentration Indices and decomposition analysis as a measure of income-related health inequality [1,9,17-20]. To our knowledge, it is the first to measure and decompose the income-related differences in adult health in urban and rural Chinese populations. Specifically, this paper seeks to understand the differing degrees of income-related health inequality in rural and in urban populations and the major factors contributing to that inequality. It estimates two major health outcome measures: (1) a subjective model assessing self-assessed health (SAH); and (2) a functional model assessing physical activity limitation. Income-related inequalities in health outcomes are calculated by Concentration Indices and presented as Concentration Curves. The contribution of socioeconomic determinants to health inequality is decomposed and quantified. Data from CHNS 2006 are used. Subsequent sections discuss the policy implications that can be drawn from this study.

## Method

### Data source

CHNS is an openly available dataset. This survey is an ongoing open cohort, international collaborative project between the Carolina Population Center at the University of North Carolina at Chapel Hill and the National Institute of Nutrition and Food Safety at the Chinese Center for Disease Control and Prevention. It was designed to examine the effects of the health, nutrition and family planning policies and programs implemented by national and local governments and to see how the social and economic transformation of Chinese society is affecting the health and nutritional status of its population. A multistage, random cluster sampling process was used to draw the sample in nine provinces in China, i.e., Liaoning, Heilongjiang, Jiangsu, Shandong, Henan, Hubei, Hunan, Guangxi and Guizhou. Counties in the nine provinces were stratified by income (low, middle and high), and a weighted sampling scheme was used to randomly select four counties in each province. In addition, the provincial capital and a lower-income city were selected when feasible. Villages and townships within the counties and urban and suburban neighborhoods within the cities were randomly selected. Although data for 2009 were available at the time of this study, health status data for that year had not yet been released. Hence, this study uses data from 2006.

Please refer to Table 1 for summary statistics on the sample. These data are drawn from CHNS 2006. The population was 50.21 per cent male and 49.79 per cent female, 29.36 per cent urban and 70.64 per cent rural. The total number of individuals surveyed was 10,182.

**Table 1:** Descriptive statistics for urban and rural populations (mean/standard deviation)

**Variable**	**Definition**	**Rural (*N*=7193)**		**Urban (*N*=2989)**	
		**Mean**	**SD**	**Mean**	**SD**
*Health variables*					
SAH	Dummy variable: 1, excellent and good health; 0 otherwise	0.593	0.491	0.594	0.491
Physical Limitation	Dummy variable: 1, having limitation coded as 1. 0 otherwise	0.072	0.259	0.081	0.272
*Demographic variables*					
Female 18-24	Dummy variable: 1, female aged between 18–24; 0 otherwise.	0.026	0.160	0.020	0.141
Female 25-34	Dummy variable: 1, female aged between 25–34; 0 otherwise.	0.076	0.265	0.065	0.246
Female 35-44	Dummy variable: 1 female aged between 35–44; 0 otherwise.	0.136	0.342	0.127	0.333
Female 45-54	Dummy variable: 1 female aged between 45–54; 0 otherwise.	0.130	0.336	0.123	0.329
Female 55-64	Dummy variable: 1 female aged between 55–64; 0 otherwise.	0.101	0.301	0.093	0.290
Female 65+	Dummy variable: 1 female aged above 65; 0 otherwise.	0.076	0.264	0.136	0.342
Male 18-24*	Dummy variable: 1 male aged between 18–24; 0 otherwise.	0.031	0.174	0.028	0.164
Male 25-34	Dummy variable: 1 male aged between 25–34; 0 otherwise.	0.079	0.269	0.052	0.222
Male 35-44	Dummy variable: 1 male aged between 35–44; 0 otherwise.	0.136	0.343	0.120	0.325
Male 45-54	Dummy variable: 1 male aged between 45–54; 0 otherwise.	0.127	0.333	0.123	0.328
Male 55-64	Dummy variable: 1 male aged between 55–64; 0 otherwise.	0.108	0.310	0.093	0.290
Male 65+	Dummy variable: 1 male aged 65 and above; 0 otherwise.	0.067	0.249	0.108	0.310
*Socioeconomic variables*					
Marital status	Dummy variable: 1 married, 0 otherwise	0.856	0.351	0.808	0.394
Job status	Dummy variable: 1 having a job, 0 otherwise	0.687	0.464	0.465	0.499
Income	Gross annual household income inflated to 2009	31,115	44,736	32,089	39,130
No education	Dummy variable: 1 no education; 0 otherwise	0.273	0.446	0.157	0.364
Pri and Sec education	Dummy variable: 1 primary and secondary education; 0 otherwise	0.554	0.497	0.371	0.483
High school education	Dummy variable: 1 high school and technical school education; 0 otherwise	0.151	0.358	0.342	0.474
University education and above*	Dummy variable: 1 university education and above; 0 otherwise	0.022	0.145	0.130	0.336
Province Liaoning	Dummy variable: 1 Liaoning, 0 otherwise	0.113	0.316	0.091	0.288
Province Heilongjiang	Dummy variable: 1 Heilongjiang, 0 otherwise	0.099	0.299	0.107	0.310
Province Jiangsu	Dummy variable: 1 Jiangsu, 0 otherwise	0.118	0.323	0.117	0.321
Province Shandong	Dummy variable: 1 Shandong, 0 otherwise	0.106	0.308	0.112	0.316
Province Henan	Dummy variable: 1 Henan, 0 otherwise	0.116	0.320	0.114	0.318
Province Hubei	Dummy variable: 1 Hubei, 0 otherwise	0.095	0.293	0.106	0.308
Province Hunan	Dummy variable: 1 Hunan, 0 otherwise	0.107	0.309	0.132	0.339
Province Guangxi	Dummy variable: 1 Guangxi, 0 otherwise	0.132	0.338	0.107	0.310
Province Guizhou*	Dummy variable: 1 Guizhou, 0 otherwise	0.115	0.319	0.112	0.316

Note: *reference groups. Gross household income is inflated to year 2009 using consumer price index.

### Statistical analysis

Income-related inequality in health is estimated using well established methods based on Concentration Curves and Concentration Indices. The method involves five basic steps: (1) estimate a model of the determinants of health outcomes, using a set of demographic and socioeconomic variables; (2) predict (indirectly) age- and sex-standardized health for each health variable, and for urban and rural respectively; (3) calculate the Concentration Indices for the actual health variables and for the standardized health variable for urban and rural populations; (4) calculate the non-demographic/socioeconomic-related inequality of health, and compare the non-demographic inequality in the rural population with that in the urban population; (5) decompose the socioeconomic factors from total health inequalities for urban and rural population respectively.

The multivariate regression models of health variables for steps (1) and (2) above are central to the methods. The health variables, i.e. SAH health and physical limitation, are binary variables. The nature of the dependent variables formally calls for a non-linear estimation. However, the disadvantage of this procedure is that certain components of the equity analysis, such as decomposition analysis, are difficult to implement and interpret. Further, studies have shown that equity measurements calculated by OLS regression do not differ importantly from the non-linear estimation [21,22]. Therefore, this paper will use OLS regression instead of non-linear regression to standardize the health variables and to decompose the Concentration Indices. Results from the Probit model are nonetheless presented in an Additional file 1: Appendix 1 in order to enable a comparison. Further, instead of using the Concentration Indices, the Erreygers Concentration Index, which has recently been developed and has proved a better estimation of binary variables, will be used [16-18,23].

The following sections will discuss the statistical analysis used for each step.

#### Standardization of health variables

Standardization of the health variables was the first step, so as to enable a reasonable estimation of health inequality. It is noted that variations in health are associated with a number of factors. In the literature, these factors are usually categorized as demographic inequalities, e.g. age and sex factors, and non-demographic inequalities arising from circumstances beyond the individual’s control, e.g. economic resources and access to healthcare. Policy may be less concerned with inequalities arising from demographic factors, e.g. demographic variation, because these are usually reasonable and accepable. Therefore, a measurement of socioeconomic-related health inequality, to control for demographic differences or identify only non-demographic differences, would be desirable for policy formation. In order to measure socioeconomic-related health inequalities that reflect only non-demographic health differences, indirect standardization of health variables is used. The aim of indirect standardization is to subtract the variation in health which is driven by demographic factors or demographic variation, and capture only the health inequality driven by non-demographic factors [9].

Standardized health variables (*ŷ*_*i*_^*X*^) is obtained by a regression of actual health variables (*ŷ*_*i*_) as follows,

(1) $$y_{i}=a+\sum_{j}\beta_{j}x_{\mathrm{ji}}+\sum_{k}\gamma_{k}z_{\mathrm{zi}}+\varepsilon_{i}$$

where x_j_ are the demographic variables, i.e., age and sex; z_k_ are non-demographic variables, i.e., (the logarithm of) income, education, job status, province of residence, urban/rural residence, marital status; α,β, and γ are the parameter vectors, and ε is the error term.

The coefficients from OLS estimations are obtained from actual values of the x_j_ variables, i.e. age and sex, which are to be standardized for, and from the sample mean for z_k_ variables, which are not to be standardized, but to be controlled for. The predicted values of health indicator ŷ_i_^X^ are then obtained.

(2) $${\hat{y}}_{i}^{X}=\hat{\alpha }+\sum_{j}{\hat{\beta }}_{j}x_{\mathrm{ji}}+\sum_{k}{\hat{\gamma }}_{k}{\bar{z}}_{\mathrm{zi}}$$

Assuming a linear model, estimates of indirectly standardized health *ŷ*_*i*_^*IS*^ can be obtained by calculating the difference between actual health (y_i_) and standardized health (*ŷ*_*i*_^*X*^), plus the sample mean ( $\bar{y}$)

(3) $${{\hat{y}}_{i}}^{\mathrm{IS}}=y_{i}-{\hat{y}}_{i}^{X}+\bar{y}$$

Rearranging the equation (3),

(4) $${{\hat{y}}_{i}}^{\mathrm{IS}}=y_{i}-\sum_{j}{\hat{\beta }}_{j}\left(x_{\mathrm{ji}}-{\bar{x}}_{j}\right)$$

Equation (4) shows that standardization will subtract the variation in health driven by demographic factors from actual health. Therefore, the distribution of ŷ^IS^ across income can be interpreted as the health status we expect to observe in an individual, irrespective of differences in the distribution of demographic characteristics.

#### Measuring income-related health inequality using concentration curves

The Concentration Index has been used in many studies to quantify the degree of socioeconomic-related inequality in health [2,24-26]. It quantifies the degree of socioeconomic-related inequality in a health variable. There are many ways to express the Concentration Index. The most convenient for the purpose of this research is:

(5) $$CI=\frac{2}{\mu }\mathrm{cov}\left(h_{\mathrm{it}},R_{i}^{t}\right)$$

Where *i* represents the individual, *h*_*i*_ is the health variable, *R* is the individual’s living standard ranking, *μ* is the mean of the health variable in the population, and *t* is the year. If there is no socioeconomic-related inequality, the index is zero. A positive value indicates a pro-rich inequality, and a negative value indicates a pro-poor inequality.

However, recent studies have suggested that there are some limitations on the Concentration Index. Wagstaff [19] has found that if the health variable of interest is binary, taking the value of 0 or the value of 1, then the bounds of the Concentration Index depend on the mean of the health variable. Therefore, this paper uses the recently introduced Erreygers Concentration Index, which is more suitable for the binary nature of the variables and the purpose of this study [17].

Erreygers proposed a revised calculation of the Concentration Index for health.

(6) $$E\left(h\right)=\frac{4\mu }{\left(b_{n}-a_{n}\right)}C\left(h\right)$$

Where b_n_ and a_n_ represent the max and min of the health variable (*h*), *μ* is the mean of the health variable in the population, and *C* (*h*) represents the Concentration Index specified in (5).

The range of the Erreygers Concentration Index is from −1 to 1. A positive value indicates a pro-rich inequality, meaning that the health variable is more concentrated among the better-off. A negative value indicates a pro-poor inequality, meaning that the health variable is more concentrated among the poor. The magnitude of the concentration index reflects the strength of the relationship between income and health variable. For example, an index of −0.7 indicates that the health variable is concentrated among the poor, and the health variable demonstrates a pro-poor inequality. Compared with an index of −0.1, an index of −0.7 indicates a more pronounced pro-poor inequality for the health variable. Similarly, an index of 0.7 indicates a pro-rich inequality; compared with an index of 0.1, an index of 0.7 indicates a more pronounced pro-rich inequality for the health variable assessed. 

Regression-based decomposition analysis helps to capture the contribution of each individual factor to income-related health inequality 9:159 [27]. The Erreygers Concentration Index can be decomposed by transforming the health variable $h_{i}=\left(h_{i}-a_{h}\right)/\left(b_{h}-a_{h}\right)$*.* Therefore, the Erreygers index differs from the decomposition of *C* by the multiplication by 4 and μ_h_. The equation is as follows.

(7) $$E=4\left[\beta \mu_{y}C_{y}+\sum_{j}\gamma_{j}\mu_{\mathrm{zj}}C_{\mathrm{zj}}+\sum_{k}\delta_{k}\mu_{\mathrm{xk}}C_{\mathrm{xk}}\right]$$

Where μ is the mean, *j* represents a vector of a set of variables *z*_*j*_, *k* represents a vector of variables *x*_*k*_, *γ* represents the coefficient of the variable z, *δ* represents the coefficient of the variable x, *C* is the Concentration Index for x, and *GC* is the generalized Concentration Index for the residual.

Another critical problem arising from calculation of the Concentration Index is the ranking indicator of the livings standard measurements. Studies have found that repetitive values of the ranking variables, i.e. two of more observations have the same values of the living standard variables, may bring instability for the calculation [28,29]. With random sorting, when a number of observations have the same value of the living standard variable, they are assigned different values of living standard-related fractional rank. Using this approach for a dataset with multiple repetitive values of the living standard variable may lead to a fictitious ranking of individuals, hence affecting the results of the Concentration Index. Specifically, Chen and Roy [28] have found that sorting observations with ascending order in the health outcome produces the upper boundary of the Concentration Index; and sorting the observations with a descending order in the health outcome produces the lower boundary of the Concentration Index. In this paper, we have sorted the data both in ascending and descending order to test the accuracy of the Erreygers Indices, and to obtain the boundaries of Erreygers Indices. The results suggest that no change is observed in terms of the value of the indices. A possible explanation, as also suggested by Chen and Roy [28], may be that individuals whose health outcomes do not deviate substantially from those with same values of the living standard variable. Hence, the estimations of Erreygers Indices in this paper are close to or the same as the true value of the Erreygers Index.

### Variable specifications

#### Dependent variables: health variables

This paper uses self-assessed health (SAH) as the dependent variable. Although SAH is a subjective measure of individual health, previous studies show that SAH is highly correlated with subsequent mortality, even when controlling for more objective health measurements [11,20,30]. In order to measure an individual’s self-assessed health status, individuals are asked: “Right now, how would you describe your health compared to that of other people of your age: excellent, good, fair, or poor?” Following a standard method, a new variable is constructed with two categories, collapsing the two lowest categories (fair and poor) [11,21]. The new SAH variable has a value of 1 if SAH is excellent or good, and otherwise of 0.

This paper also uses a functional measurement, that of physical activity limitation, as another indicator. As with SAH, this is defined as a binary variable that equals 1 if the respondent has been physically restricted and unable to perform daily activities for the past three months, and otherwise equals 0. Respondents are asked: “During the past three months have you been unable to carry out normal activities and work or studies due to illness?”

#### Independent variables

Age and gender interaction are allowed in this study as demographic variables. We categorized 12 groups: females aged 18–25, 25–34, 35–44, 55–64, and 65 and above; males aged 18–24, 25–34, 35–44, 45–54, 55–64, and 65 and above. 18-24 year-old males are the reference group.

Socioeconomic variables used in this paper are as follows. Household income inflated to 2009 using consumer price index is used as the income variable. Education is categorized by four groups: no education, primary and secondary education, high school and technical school education, and university education and above. University education and above is used as the reference group. Job status, marital status, insurance status, urban and rural residence, and province of residence are also included among the socioeconomic variables. For the province variable, the province of Guizhou is set as the reference group. Whether the respondent is treated as an urban resident or a rural respondent depends on his/her registration status as on his/her ID booklet (Hukou).

Table 1 shows the descriptive statistics for these variables.

## Results

### Descriptive analysis by urban and rural populations

Table 1 presents descriptive results for the urban and rural populations in the total sample. Urban respondents have similar self-assessed health, but more physical limitations compared with rural respondents. In terms of the demographic structure of the sample, the urban population has a much higher proportion of respondents who are above 65 years old, while the rural population has a higher proportion of respondents in other age groups. Moreover, urban respondents are more likely to have received high school and university education and are wealthier compared with the rural population. In terms of other factors, the average rates of those reporting themselves as “married” and “employed” are higher for rural than for urban respondents.

**Figure 1:**
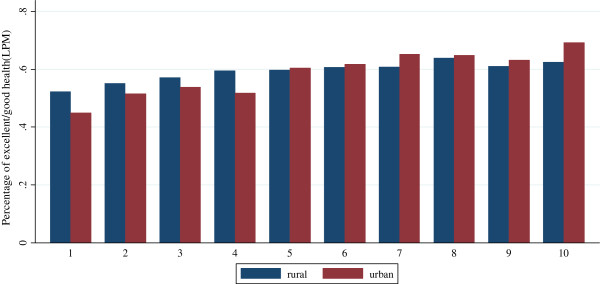
Standardized SAH (excellent and good health = 1, fair and poor health = 0) for urban population and rural population by income deciles in 2006.

**Figure 2:**
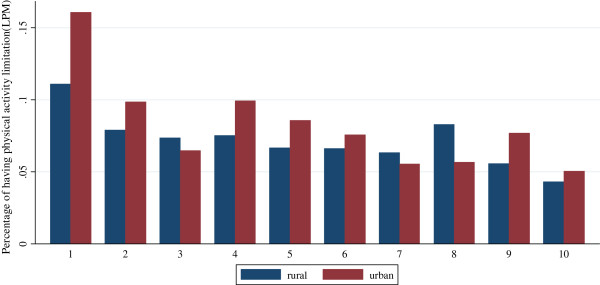
Standardized physical activity limitation for urban population and rural population by income deciles in 2006.

Figure 1 and Figure 2 show the reporting rates for SAH and physical activity limitation (standardized by age and gender) by income deciles for urban and the rural populations respectively. The rich are more likely to report their health status as excellent/good, and are less likely to report physical activity limitation. Such inequality is more pronounced for the urban population compared with the rural population.

### Determinants of health outcomes

Table 2 presents the OLS coefficients of the linear probability model. These estimates are used to calculate and decompose the Concentration Indices of the SAH and of physical activity limitation. The F test confirms the joint significance of the coefficients of all independent variables. Regarding the supposed association between income, education, and occupation types, a very low degree of correlation is found. Computation of the variance inflation factors (VIF) indicates that multicollinearity is not a problem (VIF = 2.01). A Ramsy RESET test is performed, indicating that the models showed no specification problems. As mentioned in the previous section, the nature of the variables calls formally for a non-linear estimation. Previous studies have shown that equity measurements calculated by OLS regression do not differ significantly from the non-linear estimation, and the results from this study have also proved this [21,22]. In order to be interpretable, only OLS coefficients are calculated and presented in the paper, while results from Probit models are presented in an Additional file 1: Appendix 1 in order to enable a comparison.

**Table 2:** OLS results for SAH and physical activity limitation

	**SAH (1=excellent or good, 0=fair or poor)**		**Physical Limitation (having limitation = 1, no limitation = 0)**	
	**Rural**	**Urban**	**Rural**	**Urban**
Age and gender (ref = m18-24)				
f18-24	0.1825***	0.2293***	0.0013	−0.0384
f25-34	0.1174***	0.1714***	−0.0067	−0.0238
f35-44	0.1174***	0.1259***	−0.0022	−0.0171
f45-54	0.0305	0.0135	−0.0084	−0.0097
f55-64	−0.0753***	−0.0968**	0.0093	0.0301
f65+	−0.2258***	−0.1685***	0.0326**	0.08***
m25-34	0.1598***	0.0652	−0.0196	−0.0216
m35-44	0.0391	0.0661*	−0.0122	−0.0212
m45-54	−0.0393	−0.0513	−0.002	−0.0394*
m55-64	−0.2***	−0.1497***	0.0184	0.0184
m65+	−0.255***	−0.1968***	0.0583***	0.0538**
Income (lg)	0.014**	0.0376***	−0.0077**	−0.0048
Marital Status (1 = married)	−0.0165	0.0019	0.0067	−0.0187
Job status ( 1 = having a job)	0.038***	0.0418*	−0.0374***	−0.0306**
Education level (ref = uni edu and above)				
No edu	−0.132***	−0.0301	0.0471**	0.0902***
Pri and sec edu	−0.0633	−0.0313	0.0202	0.0224
High school	−0.0131	−0.0042	0.003	0.0088
Regions (ref= Province Guizhou)				
Province Liaoning	0.0555**	0.0049	0.0042	0.0279
Province Heilongjiang	0.0869***	0.002	−0.0032	0.0569***
Province Jiangsu	0.0524**	0.1146***	0.0058	0.0082
Province Shandong	0.0974***	0.0904**	−0.0222*	−0.0032
Province Henan	−0.006	0.0072	−0.011	0.0004
Province Hubei	0.0064	0.0152	0.0325**	0.0073
Province Hunan	0.006	0.0406	0.0128	0.038*
Province Guangxi	−0.1207***	−0.1098***	0.0303**	0.0316
*Constant*	0.511***	0.2233**	0.1342***	0.1073*
Number of obs	7062	2923	7062	2923
F( 25, 7036)	42.36	15.04	8.47	7.96
Prob > F	0	0	0	0
R-squared	0.1308	0.1149	0.0292	0.0643
Adj R-squared	0.1277	0.1073	0.0258	0.0562

p < 0.01***, p < 0.05**, p < 0.1*.

As expected, a gradient in SAH by age was observed. An increase in age was associated with a deterioration in SAH. In particular, the rural population aged 65 and above has a lower probability of reporting excellent/good health compared with their urban counterparts. The impact of income on SAH was higher for the urban population than for the rural population. Having a job also increases the likelihood of reporting excellent/good health. Interestingly, the rural residents of the provinces of Liaoning, Heilongjiang, Jiangsu, Shandong and Hunan showed an increased likelihood of reporting excellent/good health compared with rural residents of other provinces.

Age is positively associated with reporting physical activity limitation. The impact of educational attainment on health is also significant. Those with no education are more likely to be physically restricted; such an impact is higher for the urban population than for the rural population. Further, those who have a job are less likely to report physical activity limitation.

### Income-related inequality in health outcomes

**Figure 3:**
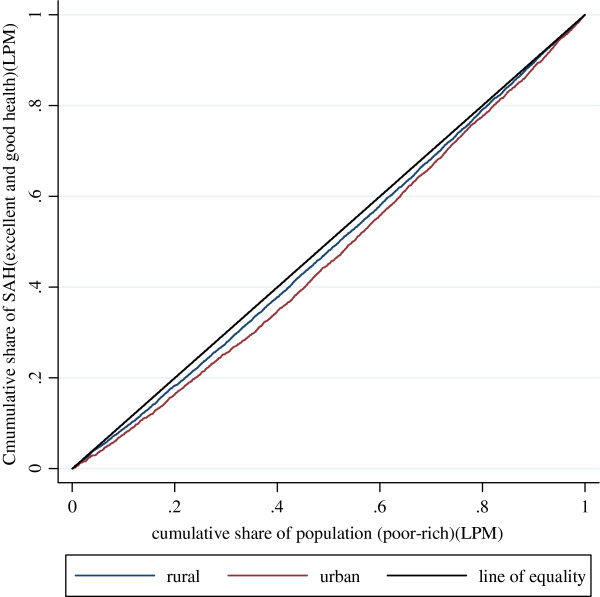
Concentration Curves for standardized SAH for urban population and rural population in 2006 (Linear Probability Model).

**Figure 4:**
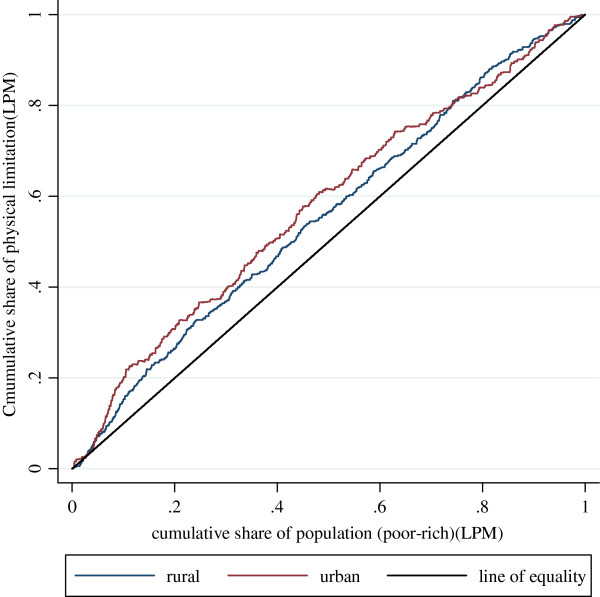
Concentration Curves for physical activity limitation for urban population and rural population in 2006 (Linear Probability Model).

Figure 3 and Figure 4 show the concentration curves for the standardized health variables, which illustrate the share of health by cumulative proportions of individuals in the population ranked from the poorest to the richest. The two key health variables are standardized by the interaction of age and gender using the indirect standardization method specified in 2.2.1. Table 3 shows the Erreygers Concentration Index (EI), non-demographic inequality, and the percentage of non-demographic inequality contributing to the total EI for urban and rural populations respectively.

**Table 3:** Erreygers’ Concentration Indices of SAH and physical activity limitation (OLS)

	**SAH**		**Physical limitation**	
	**Rural**	**Urban**	**Rural**	**Urban**
EI	0.135	0.182	−0.043	−0.060
SE (EI)	0.017	0.024	0.008	0.013
Non-demographic inequality	0.065	0.139	−0.034	−0.047
Percentage of non-demographic inequality	48.19%	76.47%	77.78%	79.07%

As shown in Table 3, the EI indicated that the rich were more likely to report excellent/good health and less likely to report physical activity limitation. Some interesting findings come from the inequality levels between urban and rural populations. Although one might assume that the urban population would have a more equal distribution of health across wealth than the rural, given some evidence demonstrated by the existing literature, the empirical results show different findings. Table 3 reports the estimates of income-related inequality indices using the Erreygers’ method (EI) for the urban and rural populations respectively. The EIs for the rural population and the urban population for SAH were 0.135 and 0.182 respectively. The indices suggest that the urban poor have a higher risk of suffering from poor health than the rural poor, as reported by their own perceptions of their health status. The EI for physical activity limitation is −0.043 for the rural population and −0.060 for the urban population, which indicates that the degree to which poverty equates with physical activity limitation is higher for the urban population compared with the rural population. Results from Probit Model are presented in Additional file 2: Appendix 2.

The indices are verified by the presentation of Concentration Curves in Figure 3 and Figure 4. The blue curves represent the rural population and the red curves the urban population. If the curves coincide with the 45-degree line of equality, all respondents, irrespective of their economic status, have the same health outcomes. If, as is more likely in this case, the curves lie above/below the 45-degree line, inequalities in health variables favor the poor/rich; such inequalities are pro-poor/pro-rich. The further the curve lies from the 45-degree line, the greater the degree of inequality in the health variable across quintiles of economic status. In Figure 3, the urban curve lies below the line of equality and below the rural curve, indicating that the urban population has a higher level of inequality favoring the rich than the rural population. In Figure 4, the urban curve lies above the line of equality and above the rural curve, indicating a more pronounced inequality in favor of the poor for the urban population compared with the rural population.

Table 3 also reports for the estimates of inequality indices that are driven mainly by non-demographic/socioeconomic factors. Results show that, for the urban population 76.47 per cent of the inequality for SAH and 79.07 per cent of the inequality for physical activity limitation is socioeconomic-related inequality. This suggests that, for the urban population, age and gender accounted for a relatively low share of income-related inequality. For the rural population, 48.19 per cent of income-related inequality in SAH and 77.78 per cent of inequality in physical activity limitation are driven by socioeconomic-related factors such as economic resources and education levels. These results indicate that a large percentage of existing income-related inequalities in SAH and physical activity limitation are potentially driven by non-demographic/socioeconomic-related factors.

### Explaining health inequalities

**Figure 5:**
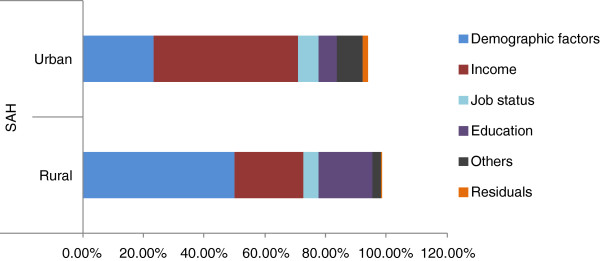
Decomposition of SAH.

**Figure 6:**
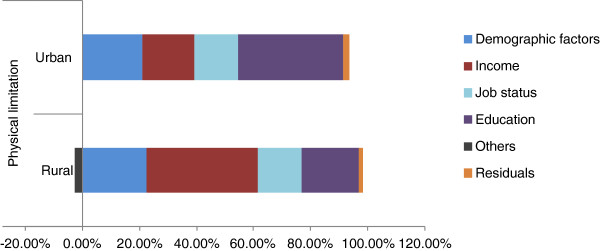
Decomposition of physical activity limitation.

The concentration index results suggest that the level of inequality in terms of health status is higher for the urban population compared with the rural population. In order to investigate this issue further, decomposition analysis is used to estimate the contribution of individual factors to total inequality. Table 4 presents the results of the decomposition analysis based on OLS regressions, indicating the contribution of individual factors to total income-related inequalities (EI). Figure 5 and Figure 6 present the individual factors. A decomposition analysis based on the Probit model is presented in an Additional file 3: Appendix 3.

**Table 4:** Decomposition results (OLS)

	**CI**		**SAH (1 = excellent or good, 0 = fair or poor)**				**Physical activity limitation**			
			** Rural**		** Urban**		** Rural**		** Urban**	
	**Rural**	**Urban**	**Contribution**	**%Contribution**	**Contribution**	**%Contribution**	**Contribution**	**%Contribution**	**Contribution**	**%Contribution**
EI			0.135		0.182		−0.043		−0.060	
Residual			0.001	0.40%	0.004	1.98%	−0.001	1.46%	−0.001	2.22%
Age and gender (ref = m18-24)										
f18-24	0.198	−0.045	0.005	3.34%	−0.001	−0.61%	0.000	−0.07%	0.000	−0.33%
f25-34	0.153	0.155	0.005	3.49%	0.005	2.59%	0.000	0.70%	−0.001	1.17%
f35-44	0.099	0.114	0.006	4.38%	0.006	3.36%	0.000	0.23%	−0.001	1.33%
f45-54	0.035	0.021	0.001	0.37%	0.000	0.06%	0.000	0.23%	0.000	0.17%
f55-64	−0.053	−0.019	0.001	1.04%	0.001	0.28%	0.000	0.46%	0.000	0.33%
f65+	−0.286	−0.072	0.015	11.42%	0.005	2.70%	−0.002	5.10%	−0.002	3.84%
m25-34	0.109	0.120	0.005	3.41%	0.002	0.99%	−0.001	1.39%	−0.001	1.00%
m35-44	0.076	0.064	0.002	1.11%	0.002	1.10%	−0.001	1.16%	−0.001	1.00%
m45-54	0.020	0.070	0.000	−0.22%	−0.002	−0.83%	0.000	0.05%	−0.001	1.83%
m55-64	−0.114	−0.087	0.007	5.49%	0.004	2.09%	−0.001	1.62%	−0.001	0.83%
m65+	−0.309	−0.221	0.022	16.24%	0.021	11.78%	−0.005	11.58%	−0.006	9.84%
ln(income)	0.056	0.058	0.031	22.77%	0.086	47.27%	−0.017	38.92%	−0.011	18.35%
Marital Status	0.013	0.044	−0.001	−0.59%	0.000	0.17%	0.000	−0.70%	−0.003	4.50%
Job status	0.064	0.161	0.007	4.97%	0.013	6.88%	−0.007	15.29%	−0.009	15.18%
Education level (ref = uni edu and above)										
No edu	−0.181	−0.356	0.026	19.36%	0.007	3.69%	−0.009	21.55%	−0.020	33.36%
Pri and sec edu	0.004	−0.113	−0.001	−0.44%	0.005	2.92%	0.000	−0.46%	−0.004	6.34%
High school	0.229	0.141	−0.002	−1.33%	−0.001	−0.44%	0.000	−0.93%	0.002	−2.84%
Regions (ref = Province Guizhou)										
Province Liaoning	0.043	0.180	0.001	0.82%	0.000	0.17%	0.000	−0.23%	0.002	−3.17%
Province Heilongjiang	−0.073	0.133	−0.003	−1.85%	0.000	0.06%	0.000	−0.23%	0.003	−5.34%
Province Jiangsu	0.232	0.240	0.006	4.30%	0.013	7.04%	0.001	−1.39%	0.001	−1.50%
Province Shandong	−0.009	−0.120	0.000	−0.30%	−0.005	−2.70%	0.000	−0.23%	0.000	−0.33%
Province Henan	−0.071	−0.071	0.000	0.15%	0.000	−0.11%	0.000	−0.93%	0.000	0.00%
Province Hubei	−0.030	−0.189	0.000	−0.07%	−0.001	−0.66%	0.000	0.93%	−0.001	1.00%
Province Hunan	0.018	−0.023	0.000	0.00%	−0.001	−0.28%	0.000	−0.23%	−0.001	0.83%
Province Guangxi	−0.011	−0.186	0.001	0.52%	0.008	4.57%	0.000	0.46%	−0.002	4.00%

The first and second columns in Table 3 show the Concentration Indices for the distribution of the independent variables, e.g. income, age and sex, marital status, etc., across income for rural and urban respondents respectively. The other columns show the contribution and percentage contribution of the individual factors to the total inequality indices for each variable and separately for the urban and the rural populations. For the rural population, the elderly, i.e. respondents above 55 years old, and those with no education are more likely to be in the low-income group. For the urban population, those with no education or with primary and secondary education only are more likely to be among the low-income groups. The better-off are more likely to have high school education and above.

The decomposition analysis, which explains the contribution of individual factors to income-related inequality, reveals some interesting findings in the comparison between rural and urban. Income, demographic features and education are the major factors contributing to inequalities. For the rural population, in terms of SAH, demographic factors contribute 50.06 per cent to total inequality, while income contributes 22.77 per cent, and education contributes 17.58 per cent. This indicates that approximately half of income-related health inequalities for the rural population are driven by demographic factors, i.e., age and gender. Further, the contribution of age and gender effects to total inequality is higher for the rural population compared to the urban population.

In terms of physical activity limitation variable, decomposition results show that demographic factors contribute 22.45 per cent to total inequality for the rural population and 21.01 per cent for the urban population. This suggests that inequality in terms of a large share of inequality for physical activity limitation is driven by non-demographic variables.

It is interesting to look at the contribution of socioeconomic-related inequalities. Figure 5 and Figure 6 show the decomposition results for SAH and physical activity limitation. The results suggest that higher-income earners are both more likely to have higher levels of education and are more likely to report excellent/good health. Further, the influence of educational attainment on pro-rich inequality is higher for the rural population compared with the urban population.

The physical activity limitation variable also reveals some interesting findings. The most important factors relating to inequality are demographic factors, income, job status and educational attainment. Results suggest that high-income earners are both well educated and less likely to have physical activity limitation. It is worth pointing out that, for the urban population, education is the most salient contributor to inequality, at approximately 40 per cent. Job status and income are the other two important factors contributing to the urban inequality indices.

## Conclusion and discussion

Policies have treated urban and rural areas in China differently. A byproduct of China’s rapid development is growing differentiation between urban and rural social and economic life. The link between social inequality and health disparity provides a particularly useful line of inquiry into the issue of urban/rural disparity. It is critically important to understand the variation in health across income groups, and in particular to take into account the effects of urban/rural residence on the degree of income-related health inequalities. This paper first compares the average health status of rural and urban populations. It then measures and compares the degree of income-related health inequalities of urban and rural populations. Factors associated with inequalities are quantified in order to illuminate the dynamic of individuals’ health and socioeconomic status for urban and rural populations respectively.

Specifically, this paper reveals some compelling new findings. The study shows that urban respondents have similar self-assessed health, but more physical limitations compared with rural respondents. Income-related health inequalities are more pronounced for urban populations as compared with rural populations. These results contradict some earlier studies, but are consistent with others. A number of the earlier studies found that living in a rural area increased the possibility of reporting poor health and that the urban population were healthier compared with the rural population [14,31]. However, a few other studies demonstrated different findings. For instance, Van de Poel et al. [32] found that urban residents were more likely to have a higher incidence of chronic diseases, and that obesity and hypertension rates were more prevalent in urban China than in rural China. A possible explanation suggested by the authors was that the rapid environmental, economic and social changes that followed urbanization increased the prevalence of major risk factors for chronic disease. The increasing urbanization and development may change the geographical distribution of non-communicable diseases. Further, urban areas in low- and middle-income countries, such as China, were moving through a rapid nutritional transition towards western-style diets dominated by more processed foods and a higher fat content. Increasing urbanization also led to equally rapid shifts towards more sedentary occupations through the acquisition of new technology and transitions away from an agricultural economy, which may also cause health problems [15,16,32].

The total differential decomposition allows us to disentangle causes of changing inequality. Possible policy implications can be drawn from these results. The empirical results suggest that, for the rural population, the young, the better-off, and the educated are less likely to suffer from ill health. Similarly, for the urban population, income contributes strongly to inequality. Apart from income, educational attainment and job status also make a positive contribution to total inequalities. The study also finds that, for the urban population, 76.47 per cent to 79.07 per cent of inequalities are driven by socioeconomic-related factors. Income, job status and educational attainment each appear to have a prominent influence on inequality. For the rural population, 48.19 per cent to 77.78 per cent of inequality can be explained by socioeconomic-related factors, among which income and educational level are the most important factors. These findings are consistent with some of the previous studies. The role of income is notable. Wagstaff et al. [1] found that income played an important role in child malnutrition in the 1990s in Vietnam. They suggested that, although rising incomes reduced malnutrition and hence reduced average malnutrition, rising incomes also directly increased relative inequality in malnutrition, magnifying the inequality in malnutrition attributable to income inequality. As also indicated by the 2008 National Health Service Survey [33], income level was a major determinant of health outcomes. Being poor and lacking healthcare coverage often prevented people from seeking care [6]. Hence, promoting health equality and providing support for the poor and for those with special health needs are important strategies for maintaining sustainable development and alleviating poverty. As the present study has indicated an urban disadvantage with respect to health inequalities, there is certainly a need, if equality in health is to be realized, for better facilities in urban areas and to provide the urban poor with support.

The contribution of education is also important. Previous studies found that educational level made an important contribution to total inequality, and that its effect was even more important in some cases than the “pure income effect”. Anson and Sun [31] suggested that the association between education and income in China resembled the patterns documented in industrial societies. Level of education, higher income and occupational status were all significantly related to health. Similar results were reported by Costa-i-Font et al. [23], who examined socioeconomic inequalities in obesity and found that education was an important determinant in explaining obesity. The possible explanation given by Costa-i-font et al. was that education helped to convey unobserved effects such as knowledge transfer, which enabled people to be more health-conscious. Meanwhile, the translation of income into better living environment and healthy food may be as efficient as other effects such as knowledge transfer, presumably identified by the education treatment variable [23]. Hence, they suggested that government should coordinate a number of policies including promoting or subsidizing knowledge communication on healthy life styles. These implications are relevant and applicable in the Chinese context. Since physical exercise, healthy diet and sleeping habits may have an influence on the behavior of certain low-income groups that are more oriented to unhealthy lifestyles, the prevention of certain unhealthy habits through knowledge-related activities directed especially at low-income individuals is likely to have desirable effects in reducing income-related inequalities in health [23,34].

It is worth pointing out that the healthcare systems in rural and urban areas may also affect the inequalities in health outcomes. The gap in distribution of health resources between urban and rural areas has been narrowed in the past a few decades, and substantial progress has been made in rural areas [35]. For the past decade, the Chinese government has been making concerted efforts to build new primary and secondary health facilities in rural areas in order to improve access to basic medical care [36]. The New Operative Medical Insurance Scheme was initiated in 2003 to protect the rural population from disease and ill health [37]. On the other hand, the urban health system, despite absorbing a disproportionately large share of total health subsidies, has been criticized as plagued by inefficiency and low quality, by an overly concentrated use of services on tertiary care and by over-prescribing and over-use of health service, all of which may lead to health inequality and other health problems [35,38-41]. These problems may give rise to access and affordability issues, thus damaging the population’s health, particularly that of low-income groups. The Chinese government has apparently noticed these issues and is in the process of improving its healthcare sector in order to tackle inequalities. More primary healthcare facilities have been built. New health insurance schemes, such as the New Cooperative Medical Insurance Scheme and the Urban Residents Medical Insurance Scheme, have been introduced in order to target the rural population and the urban poor [36,38,40,42]. The government is moving in the right direction to combat inequality, but how well these policies have been implemented and how effective they will be is yet to be shown.

This study has its own limitations, although it is among the first to provide evidence from China on urban/rural disparity in income-related adult health. The first concerns the dataset. The dataset used is probably by far the most comprehensive ever used in studying health inequality in the Chinese context; however, only nine provinces were included. Most of these provinces are situated in the eastern and coastal part of China, where the levels of economic development are high. Hence, any further generalization should be made with caution. Another limitation is the variables of interest. Self-assessed health variables can be biased because of problems in reporting. If reporting differences have influenced the population equally, this will not be a problem. However, it is possible that population groups may report the variable in a systematically different way. For instance, under-reporting may be greater in rural than in urban areas. Old people may be more likely to underestimate their health status compared with young people. If this were the case, the results shown here might represent an underestimation of inequality in certain population groups. However, these are the limitations of most health outcome measurements in the absence of other possible objective variables.

## Competing interests

There are no financial or non-financial competing interests in this paper.

## Authors’ contributions

WY developed the research idea, conducted the data analysis, and drafted the paper. PK helped with the statistical procedure and finalized the manuscript. Both authors read and proved the final manuscript.

## Pre-publication history

The pre-publication history for this paper can be accessed here:

http://www.biomedcentral.com/1471-2458/12/804/prepub

## Supplementary Material

Additional file 1 **Appendix A. **Appendix 1: Probit model results of SAH and physical activity limitation. Click here for file

Additional file 2 **Appendix B. **Appendix 2: Erreygers’ Concentration Indices of SAH and physical activity limitation (Probit). Click here for file

Additional file 3 **Appendix C. **Appendix 3: Decomposition results (Probit). Click here for file
