# Supplementary material for: The less healthy urban population: income-related health inequality in China
Source: BMC Public Health. 2012 Sep 18;12:804. doi: 10.1186/1471-2458-12-804 (PMC3563496; doi:10.1186/1471-2458-12-804)
Supplement: Additional file 1 — Appendix A. Appendix 1: Probit model results of SAH and physical activity limitation. [file 1471-2458-12-804-S1.doc]

Appendix A. Appendix 1

**Probit model results of SAH and physical activity limitation**

|  | SAH (1=excellent or good, 0=fair or poor) | | Physical Limitation(having limitation = 1, no limitation = 0) | |
| --- | --- | --- | --- | --- |
|  | Rural | Urban | Rural | Urban |
| Age and gender (ref = m18-24) |  |  |  |  |
| f18-24 | 0.2091*** | 0.255*** | -0.0000429 | -0.0276 |
| f25-34 | 0.1291*** | 0.2044*** | -0.0104 | -0.0298 |
| f35-44 | 0.1273*** | 0.1385*** | -0.0053 | -0.0205 |
| f45-54 | 0.0316 | 0.0161 | -0.0107 | -0.0111 |
| f55-64 | -0.0748*** | -0.0978** | 0.0083 | 0.0252 |
| f65+ | -0.2282*** | -0.1668*** | 0.024 | 0.0565** |
| m25-34 | 0.1726*** | 0.0716 | -0.023 | -0.0254 |
| m35-44 | 0.0413 | 0.0721* | -0.0134 | -0.0225 |
| m45-54 | -0.0389 | -0.0503 | -0.0007 | -0.0387** |
| m55-64 | -0.2014*** | -0.1488*** | 0.0156 | 0.013 |
| m65+ | -0.2633*** | -0.1971*** | 0.0382** | 0.0308 |
| Income(lg) | 0.0152** | 0.0407*** | -0.0066** | -0.0048 |
| Marital Status | -0.0178 | 0.0038 | 0.0053 | -0.0138 |
| Job status | 0.0436*** | 0.0478* | -0.0338*** | -0.0354*** |
| Education level (ref = uni edu and above) |  |  |  |  |
| No edu | -0.154*** | -0.0305 | 0.065** | 0.0748*** |
| Pri and sec edu | -0.0816* | -0.034 | 0.0328 | 0.0226 |
| High school | -0.0244 | -0.0075 | 0.0122 | 0.01 |
| Regions (ref= Province Guizhou) |  |  |  |  |
| Province Liaoning | 0.0609** | 0.0077 | 0.0035 | 0.0284 |
| Province Heilongjiang | 0.0968*** | 0.0035 | -0.0064 | 0.0588** |
| Province Jiangsu | 0.0547** | 0.1246*** | 0.0036 | 0.0089 |
| Province Shandong | 0.1076*** | 0.0967** | -0.024** | -0.0039 |
| Province Henan | -0.0075 | 0.0069 | -0.0133 | -0.0028 |
| Province Hubei | 0.0079 | 0.0191 | 0.0302** | 0.008 |
| Province Hunan | 0.007 | 0.045 | 0.0125 | 0.0363* |
| Province Guangxi | -0.1345*** | -0.1156*** | 0.0264** | 0.0293 |
|  |  |  |  |  |
| *Constant* |  |  |  |  |
|  |  |  |  |  |
| Number of obs | 7062 | 2923 | 7062 | 2923 |
| LR chi2(25) | 963.18 | 354.01 | 191.24 | 174.58 |
| Prob > chi2 | 0 | 0 | 0 | 0 |
| Pseudo R2 | 0.1009 | 0.0898 | 0.0523 | 0.1077 |
| Log likelihood | -4290.08 | -1794.89 | -1733.27 | -723.05 |
