# Supplementary material for: The less healthy urban population: income-related health inequality in China
Source: BMC Public Health. 2012 Sep 18;12:804. doi: 10.1186/1471-2458-12-804 (PMC3563496; doi:10.1186/1471-2458-12-804)
Supplement: Additional file 2 — Appendix B. Appendix 2: Erreygers’ Concentration Indices of SAH and physical activity limitation (Probit). [file 1471-2458-12-804-S2.doc]

Appendix B. Appendix 2

**Erreygers’ Concentration Indices of SAH and physical activity limitation (Probit)**

|  | SAH | | Physical Limitation | |
| --- | --- | --- | --- | --- |
|  | Rural | Urban | Rural | Urban |
| EI | 0.135 | 0.182 | -0.043 | -0.060 |
| SE (EI) | 0.017 | 0.024 | 0.008 | 0.013 |
| Non-demographic inequality | 0.067 | 0.138 | -0.035 | -0.049 |
| Percentage of non-demographic inequality | 49.95% | 75.84% | 81.91% | 82.51% |
