# Supplementary material for: The less healthy urban population: income-related health inequality in China
Source: BMC Public Health. 2012 Sep 18;12:804. doi: 10.1186/1471-2458-12-804 (PMC3563496; doi:10.1186/1471-2458-12-804)
Supplement: Additional file 3 — Appendix C. Appendix 3: Decomposition results (Probit). [file 1471-2458-12-804-S3.doc]

Appendix C. Appendix 3

**Decomposition results (Probit)**

|  | CI | | SAH (1=excellent or good, 0=fair or poor) | | | | Physical Activity Limitation | | | |
| --- | --- | --- | --- | --- | --- | --- | --- | --- | --- | --- |
|  |  |  | Rural |  | Urban |  | Rural |  | Urban |  |
|  | Rural | Urban | Contribution | %Contribution | Contribution | %Contribution | Contribution | %Contribution | Contribution | %Contribution |
| EI |  |  | 0.135 |  | 0.182 |  | -0.043 |  | -0.060 |  |
| Residual |  |  | -0.002 | -1.35% | 0.001 | 0.33% | -0.002 | 4.23% | 0.001 | -1.01% |
|  |  |  |  |  |  |  |  |  |  |  |
| Age and gender (ref = m18-24) |  |  |  |  |  |  |  |  |  |  |
| f18-24 | 0.198 | -0.045 | 0.005 | 3.86% | -0.001 | -0.72% | 0.000 | 0.00% | 0.000 | -0.17% |
| f25-34 | 0.153 | 0.155 | 0.005 | 3.86% | 0.006 | 3.14% | 0.000 | 0.93% | -0.001 | 1.33% |
| f35-44 | 0.099 | 0.114 | 0.006 | 4.75% | 0.007 | 3.69% | 0.000 | 0.70% | -0.001 | 1.67% |
| f45-54 | 0.035 | 0.021 | 0.001 | 0.37% | 0.000 | 0.06% | 0.000 | 0.46% | 0.000 | 0.17% |
| f55-64 | -0.053 | -0.019 | 0.001 | 1.04% | 0.001 | 0.28% | 0.000 | 0.46% | 0.000 | 0.17% |
| f65+ | -0.286 | -0.072 | 0.016 | 11.57% | 0.005 | 2.64% | -0.002 | 3.71% | -0.002 | 2.67% |
| m25-34 | 0.109 | 0.120 | 0.005 | 3.71% | 0.002 | 1.10% | -0.001 | 1.62% | -0.001 | 1.17% |
| m35-44 | 0.076 | 0.064 | 0.002 | 1.11% | 0.002 | 1.16% | -0.001 | 1.16% | -0.001 | 1.17% |
| m45-54 | 0.020 | 0.070 | 0.000 | -0.22% | -0.002 | -0.83% | 0.000 | 0.00% | -0.001 | 1.83% |
| m55-64 | -0.114 | -0.087 | 0.008 | 5.56% | 0.004 | 2.09% | -0.001 | 1.39% | 0.000 | 0.50% |
| m65+ | -0.309 | -0.221 | 0.023 | 16.76% | 0.022 | 11.83% | -0.003 | 7.65% | -0.003 | 5.50% |
| ln(income) | 0.056 | 0.058 | 0.033 | 24.77% | 0.093 | 51.18% | -0.014 | 33.36% | -0.011 | 18.18% |
| Marital Status | 0.013 | 0.044 | -0.001 | -0.59% | 0.001 | 0.28% | 0.000 | -0.46% | -0.002 | 3.34% |
| Job status | 0.064 | 0.161 | 0.008 | 5.71% | 0.014 | 7.87% | -0.006 | 13.90% | -0.011 | 17.68% |
| Education level (ref = uni edu and above) |  |  |  |  |  |  |  |  |  |  |
| No edu | -0.181 | -0.356 | 0.030 | 22.54% | 0.007 | 3.74% | -0.013 | 29.65% | -0.017 | 27.69% |
| Pri and sec edu | 0.004 | -0.113 | -0.001 | -0.52% | 0.006 | 3.14% | 0.000 | -0.70% | -0.004 | 6.34% |
| High school | 0.229 | 0.141 | -0.003 | -2.52% | -0.001 | -0.77% | 0.002 | -3.94% | 0.002 | -3.17% |
| Regions (ref= Province Guizhou) |  |  |  |  |  |  |  |  |  |  |
| Province Liaoning | 0.043 | 0.180 | 0.001 | 0.89% | 0.001 | 0.28% | 0.000 | -0.23% | 0.002 | -3.17% |
| Province Heilongjiang | -0.073 | 0.133 | -0.003 | -2.08% | 0.000 | 0.11% | 0.000 | -0.46% | 0.003 | -5.50% |
| Province Jiangsu | 0.232 | 0.240 | 0.006 | 4.52% | 0.014 | 7.65% | 0.000 | -0.93% | 0.001 | -1.67% |
| Province Shandong | -0.009 | -0.120 | 0.000 | -0.30% | -0.005 | -2.86% | 0.000 | -0.23% | 0.000 | -0.33% |
| Province Henan | -0.071 | -0.071 | 0.000 | 0.22% | 0.000 | -0.11% | 0.000 | -0.93% | 0.000 | -0.17% |
| Province Hubei | -0.030 | -0.189 | 0.000 | -0.07% | -0.002 | -0.88% | 0.000 | 0.70% | -0.001 | 1.17% |
| Province Hunan | 0.018 | -0.023 | 0.000 | 0.07% | -0.001 | -0.28% | 0.000 | -0.23% | 0.000 | 0.67% |
| Province Guangxi | -0.011 | -0.186 | 0.001 | 0.52% | 0.009 | 4.79% | 0.000 | 0.23% | -0.002 | 3.67% |
